# Acute enhancing effect of a standardized extract of *Centella asiatica* (ECa 233) on synaptic plasticity: an investigation via hippocampal long-term potentiation

**DOI:** 10.1080/13880209.2021.1893348

**Published:** 2021-03-31

**Authors:** Yingrak Boondam, Mayuree H. Tantisira, Kanokwan Tilokskulchai, Sompol Tapechum, Narawut Pakaprot

**Affiliations:** a Department of Physiology, Faculty of Pharmacy, Mahidol University, Bangkok, Thailand; b Faculty of Pharmaceutical Sciences, Burapha University, Chonburi, Thailand; c Department of Physiology, Faculty of Medicine Siriraj Hospital, Mahidol University, Bangkok, Thailand

**Keywords:** LTP, hippocampus, learning, cognition, nootropic, herbal plant

## Abstract

**Context:**

ECa 233 is the standardized extract of *Centella asiatica* (L.) Urban. (Apiaceae). It contains at least 85% of triterpenoid glycosides and yields neuroprotective and memory-enhancing effects. However, the exact molecules exerting the effects might be triterpenic acid metabolites reproduced through gut metabolism after orally ingesting *C. asiatica*, not triterpenoid glycosides.

**Objective:**

This study demonstrates the effect of unmetabolized ECa 233 on hippocampal synaptic plasticity after directly perfusing ECa 233 over acute brain slices.

**Materials and methods:**

The brain slices obtained from 7-week-old male Wistar rats were randomly divided into 4 groups. We perfused either artificial cerebrospinal fluid (ACSF), 0.01% DMSO, 10 µg/mL ECa 233, or 100 µg/mL on brain slices, and measured the long-term potentiation (LTP) magnitude to determine the synaptic plasticity of hippocampal circuits in each group.

**Results:**

The LTP magnitude of ACSF, DMSO, 10 ug/mL ECa 233, and 100 ug/mL ECa 233 groups increased from 100% to 181.26 ± 38.19%, 148.74 ± 5.40%, 273.71 ± 56.66%, 182.17 ± 18.61%, respectively. ECa 233 at the concentration of 10 µg/mL robustly and significantly enhanced hippocampal LTP magnitude. The data indicates an improvement of the hippocampal synaptic plasticity.

**Discussion and conclusions:**

This study emphasizes the effectiveness of triterpenoid glycosides in ECa 233 on synaptic plasticity enhancement. Therefore, this study supported and complimented the known effects of *C. asiatica* extract on the enhancement of synaptic plasticity, and subsequently, learning and memory, suggesting that ECa 233 could be a promising memory enhancing agent.

## Introduction

The standardized extract of *Centella asiatica* (L.) Urban. (Apiaceae), known as ECa 233, is a white to off-white powder extract containing at least 85% of triterpenoid glycosides, of which 53.1% is madecassoside and 32.3% is asiaticoside. The remaining 15% consists of triterpenoid saponins, of which less than 1% are triterpenic acid metabolites – madecassic acid and asiatic acid (Anukunwithaya et al. 2017a). ECa 233 provides neuroprotection in both *in vitro* and *in vivo* studies. It also enhances the neurite outgrowth of neuroblastoma cells via the MEK/ERK and PI3K/Akt pathways (Wanakhachornkrai et al. 2013). Furthermore, ECa 233 promotes the survival of dopaminergic neurons in the rotenone-induced parkinsonian rats via its antioxidant activities (Teerapattarakan et al. 2018). In our recent study, 30 mg/kg dose of ECa 233 significantly enhanced memory retention of healthy normal rats. The improved behavioural performance was in line with the increase of synaptic-related proteins in the hippocampus (e.g., NR2A and NR2B subunits of *N*-methyl-d-aspartate [NMDA] receptor, postsynaptic density 95 [PSD-95], brain-derived neurotrophic factor [BDNF], and tyrosine kinase B [TrkB]) and of the late phase of hippocampal long-term potentiation (LTP) magnitude. Thus, the mechanism of synaptic plasticity enhancement by ECa 233 might be accounted by the activation of BDNF/TrkB and NMDAR signalling cascades (Boondam et al. 2019).

However, the mechanisms of *C. asiatica* extract, especially the exact active compounds of the extract that produce the memory-enhancing effect, remain unclear. Although *C. asiatica* extract mainly contains triterpenoid glycosides with very few triterpenic acid metabolites, namely, madecassic acid and asiatic acid (<1% in ECa 233), β-glycosidases from gut microbiota metabolize the triterpenoid glycosides to triterpenic acid metabolites after the oral administration of the extract (Anukunwithaya et al. 2017b; Khemawoot et al. 2018). In rats, triterpenic acid metabolites were largely found in faeces (approx. 50% of the consumed doses of the parent compounds) but scarcely in plasma and organ tissues (Anukunwithaya et al. 2017b). In humans, a large amount of these metabolites was found in the plasma according to LC-MS/MS system analysis (Songvut et al. 2019). Some studies of *C. asiatica* extract have shown that triterpenic acid metabolites have an effect on learning and memory via the neuroprotective and anxiolytic activities (Nasir et al. 2011; Sirichoat et al. 2015; Chaisawang et al. 2017). However, the exact active compounds that produce the memory-enhancing effect remain inconclusive.

In addition to the effect from the gut metabolism, the low oral bioavailability of the extract (Anukunwithaya et al. 2017b) might affect the action of ECa 233. The number of active constituents entering the brain might be lower than the effective dose; hence, the extract might take time to generate their effects, as shown in our previous study in which the extract was given orally for a month (Boondam et al. 2019). Other studies have demonstrated that the duration of oral administration ranged from 2 weeks to 2 months (Soumyanath et al. 2012; Tabassum et al. 2013; Puttarak et al. 2017; Gray et al. 2018; Ar Rochmah et al. 2019). In contrast, intravenous ECa 233 shows 100% bioavailability, and it can cross the blood–brain barrier, as observed in brain tissue accumulation (Anukunwithaya et al. 2017b; Khemawoot et al. 2018; Boondam et al. 2019). Thus, ECa 233 perfusion into the acute hippocampal slices will show the direct action of ECa 233 on the hippocampal neurons in which their synaptic plasticity is crucial for learning and memory.

To clarify and emphasize the memory-enhancing capacity of *C. asiatica* extract, this study aimed to elucidate the efficacy of ECa 233 on hippocampal synaptic plasticity. Here we mimicked the systemic administration of the extract by allowing direct ECa 233 perfusion on the acute hippocampal slice. We proposed that this method could eliminate drug absorption-influencing factors such as intestinal transporter, intestinal metabolism, gastric emptying time, and hepatic metabolism. This method would also exhibit the direct effect of parent compounds on neuronal tissue. Moreover, we assessed the LTP to observe synaptic plasticity, which is a necessary process that leads to hippocampal synapse strengthening and is also the basic mechanism of learning-and-memory formation.

## Material and methods

### Chemical and reagents

ECa 233 containing madecassoside (51% w/w) and asiaticoside (38% w/w) was obtained from Siam Herbal Innovation Ltd. in Thailand (lot number MRA 0816001). DMSO was purchased from Sigma-Aldrich Corp.

### Animals

We utilized healthy male Wistar rats aged 7 weeks and weighed 180–210 g. The animals were purchased from the National Laboratory Animal Centre, Mahidol University in Thailand. All rats were housed under the conditions of controlled humidity, temperature, and light (12 h day/night cycle) with free access to water and murine chow, consistent with the guideline for animal care and use in a scientific study. The animal ethics committee of the Faculty of Medicine Siriraj Hospital in Mahidol University had approved our study before the experimentation (approval number: COA 001/2561).

### Hippocampal slice preparation

The rats were deeply anaesthetized with isoflurane until they were unconscious, followed by quick decapitation using a guillotine. The brain was taken out rapidly and transferred immediately into 4 °C oxygenated artificial cerebrospinal fluid (ACSF; 124 mM NaCl, 3 mM KCl, 1.25 mM NaH_2_PO4, 1.30 mM MgSO_4_-7H_2_O, 26 mM NaHCO_3_, 10 mM d-glucose, and 2.4 mM CaCl_2_-2H_2_O) with sucrose solution. We then sectioned 350 μm thick transverse hippocampal slices, stored them in a nylon-mesh interface-type tissue chamber, and incubated them in carbogen (95% O_2_ and 5% CO_2_)-saturated ACSF at room temperature for at least 1 h before electrophysiological recording.

### Electrophysiological recordings

To record extracellular field excitatory postsynaptic potentials (fEPSPs), we filled an extracellular recording electrode with ACSF and positioned it at the dendritic layer of the hippocampus’ CA1 area. We then placed a tungsten stimulating electrode 1000 μm away from the recording electrode at the same dendritic layer to activate the Schaffer collateral commissural fibres. The fEPSPs were recorded using PowerLab equipment and LabChart 7 software (AD Instruments, Dunedin, New Zealand).

#### Input-output recordings

We used an input-output (I-O) protocol to determine the basal synaptic transmission for each slice. Recordings were made in response to a single constant current stimulation once every 30 s. Furthermore, we varied the intensity (30–300 μA) to gradually adjust stimulus intensities, which were then applied to the Schaffer collateral commissural fibres. The fEPSPs were recorded at the dendritic layer of the CA1 area.

#### Hippocampal LTP recordings after extracellular application of the substances

**Figure 1.**
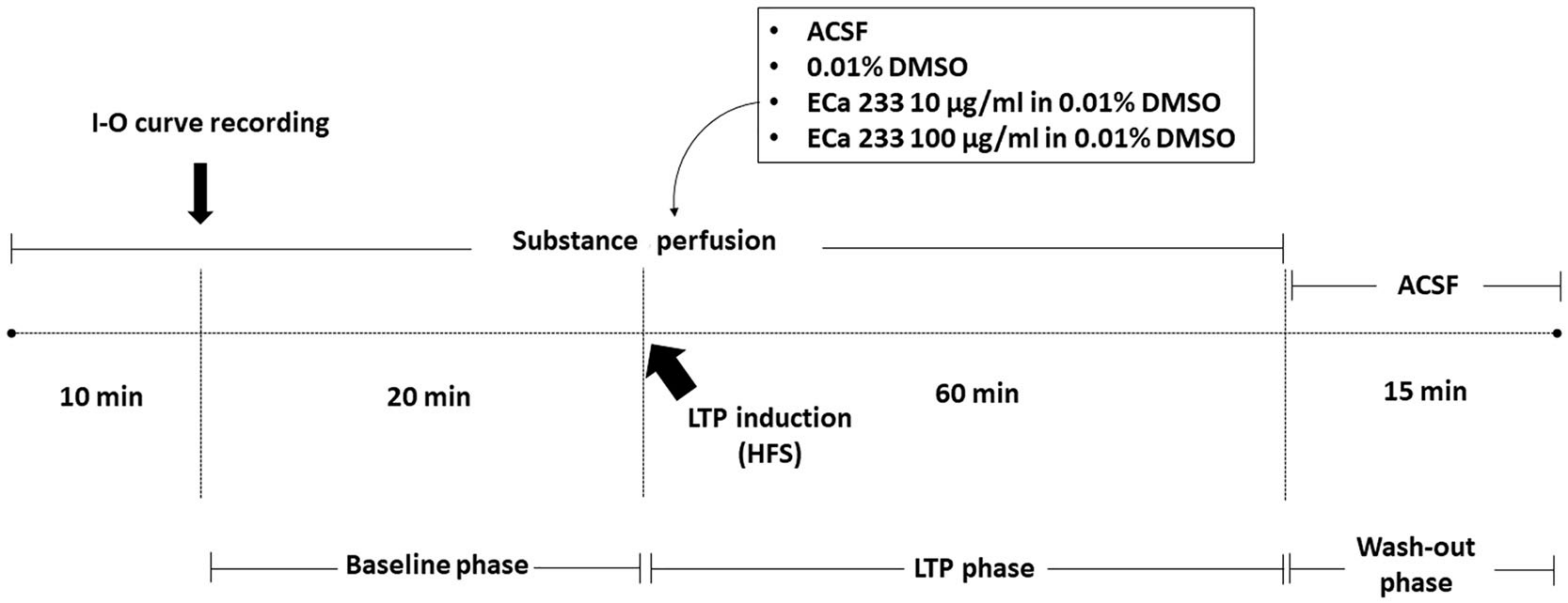
Experimental protocol. After 10 min of acute perfusion with a tested substance (ACSF, 0.01% DMSO, 10 µg/mL ECa 233 in 0.01% DMSO, or 100 µg/mL ECa 233 in 0.01% DMSO), an I-O curve was recorded (the stimulus intensities were varied from 30 μA to 300 μA). Then, the stimulus intensity of half-maximal fEPSP amplitude from the I-O curve was used for the stimulation on the hippocampal slices throughout the experiment. The baseline activity was recorded for 20 min. Then, the HFS was delivered at the same stimulus intensity to evoke LTP, and the activity was continually recorded for 60 min (LTP phase). After 60 min of recording, the solutions were reverted to ACSF, and the activity was continually recorded for 15 min (washed-out phase).

The hippocampal LTP is a cellular model of the synaptic plasticity that represents hippocampal synapse strengthening, which is the basic mechanism of learning-and-memory formation. The stimulus intensity of the half-maximal fEPSP amplitude determined from the I-O curve of each slice was used on that hippocampal slice throughout the experiment. Recordings were still made in response to a single constant current stimulation once every 30 s. After 10 min of direct perfusion at approximately 1.5 mL/min with a tested substance (ACSF, 0.01% DMSO, 10 µg/mL ECa 233 in 0.01% DMSO, or 100 µg/mL ECa 233 in 0.01% DMSO), we recorded the baseline activity for 20 min. To induce LTP, we delivered high-frequency stimulation (HFS) containing two trains of tetanic stimulation (100 Hz of 100 pulses) of the same stimulus intensity and continually recorded the neuronal activity for 60 min (LTP phase). Thereafter, we reverted the solution to ACSF and continually recorded the activity for 15 min (washed-out phase). Next, we calculated the peak slope of the rising phase of the fEPSPs (1 ms duration). Figure 1 illustrates the experimental protocol.

### Statistical analysis

Data are expressed as the mean ± SEM. Reported ‘n’ refers to the number of hippocampal slices. The results of the I-O curve were statistically evaluated using one-way analysis of variance (ANOVA), followed by Fisher’s least-significant difference (LSD) *post hoc* test. Moreover, the results of hippocampal LTP magnitude were statistically evaluated using both one-way and two-way ANOVA, followed by Fisher’s LSD *post hoc* test. All statistical data were analysed using IBM SPSS 21 (IBM, Armonk, NY, USA). Statistical significance was represented by *p* < 0.05.

## Results

### ECa 233 did not alter the basal synaptic transmission

**Figure 2.**
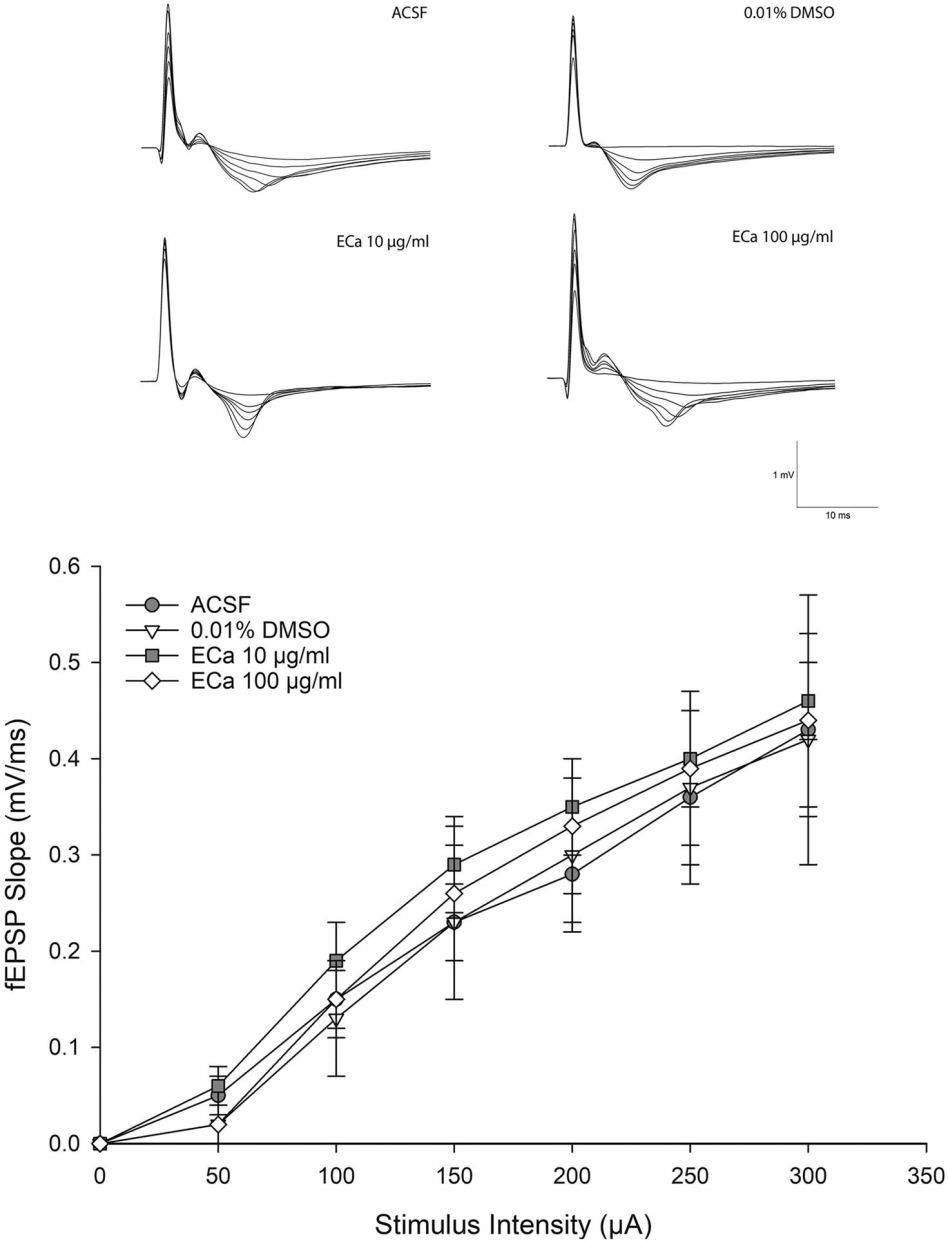
Acute effect of ECa 233 on the hippocampal basal synaptic transmission. The I-O curve represents the basal synaptic transmission. The stimulus intensities varying from 30 μA to 300 μA were applied at the Schaffer collateral pathway. The fEPSP slopes were recorded at the dendritic layer of the CA1 subregion. Data are presented as mean ± SEM (*n* = 4).

The basal synaptic transmission was evaluated by the I-O curve. The gradual increment of stimulus intensity would enhance the synaptic responses. The I-O curve recordings (Figure 2) indicated that ECa 233 did not alter the basal excitatory synaptic transmission at any concentrations compared with that in each group [F_(3,11)_ = 0.730, *p* = 0.562]. Hence, ECa 233 did not alter the efficacy of electrical impulse transmitted by hippocampal neurons at the basal state. Therefore, these results could extrapolate to the LTP results that the enhanced expression of LTP was not a subsequent outcome of the increased basal neural activity.

### ECa 233 enhanced the hippocampal synaptic plasticity

**Figure 3.**
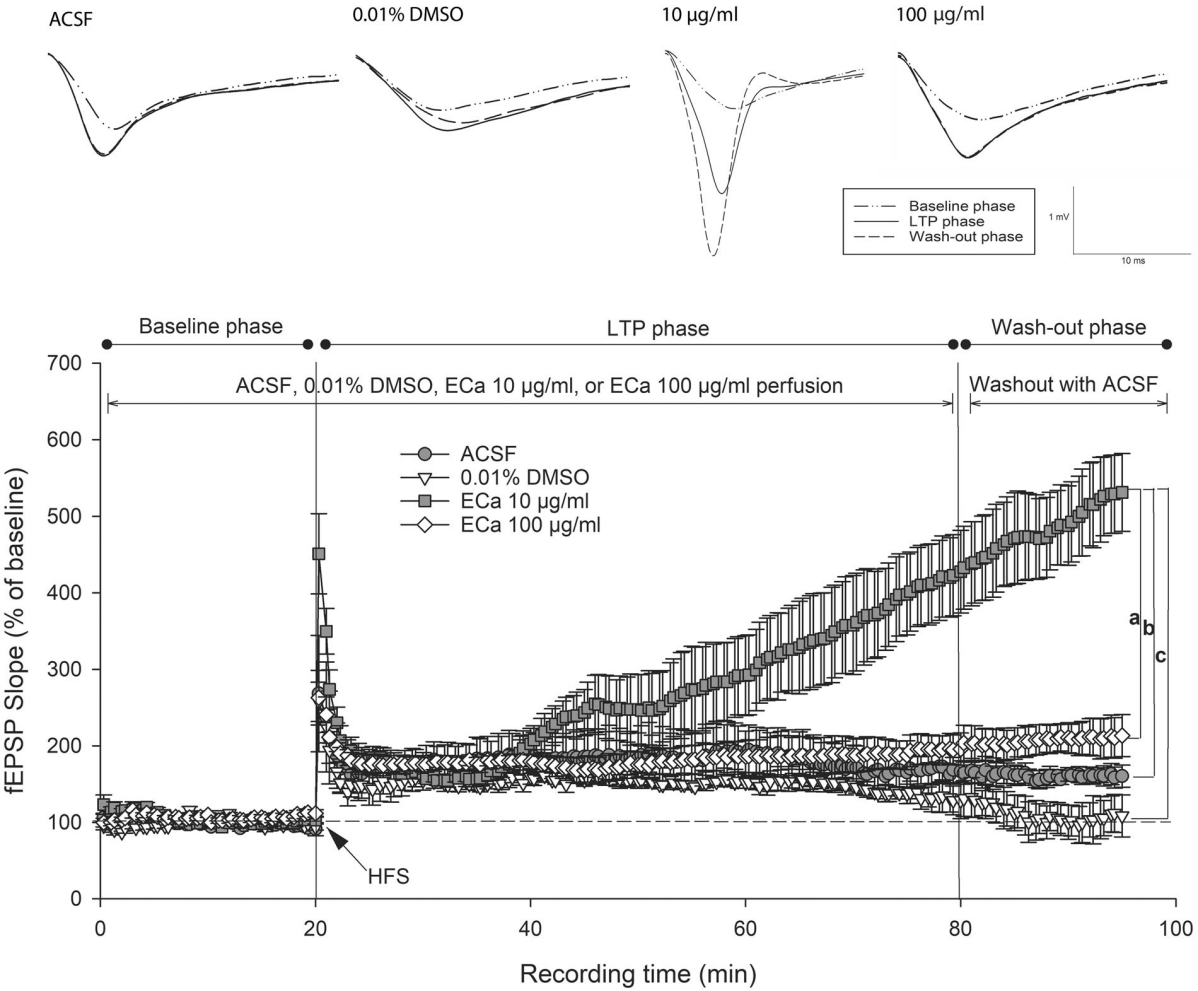
Enhancement of hippocampal long-term potentiation magnitude after acute treatment with different ECa 233 concentrations. Data are presented as mean ± SEM (n = 4). ^a^*p* = 0.041 compared with the ECa 100 µg/mL group; ^b^*p* = 0.037 compared with the ACSF group; and ^c^*p* = 0.008 compared with the 0.01% DMSO group.

**Figure 4.**
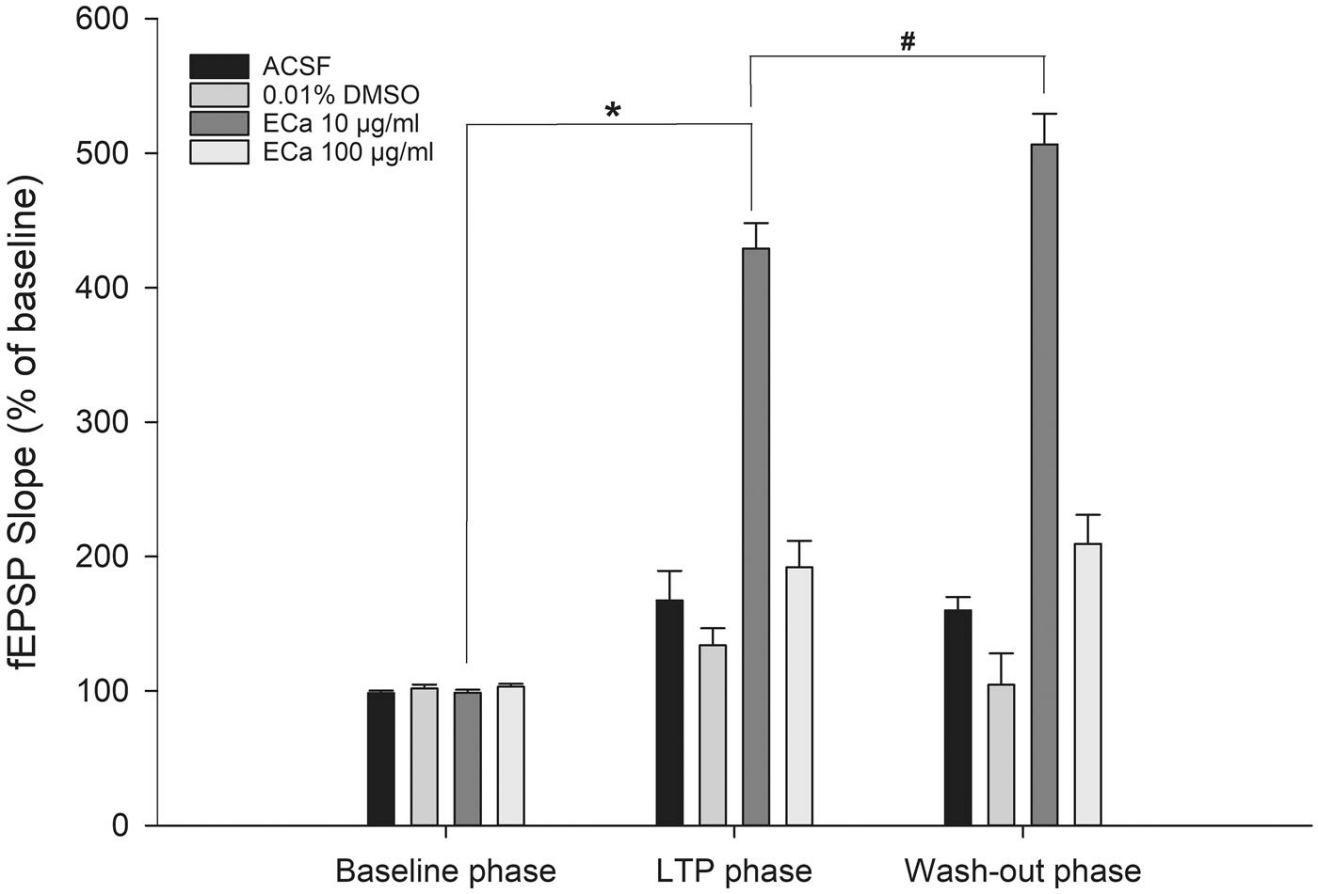
Acute effect of ECa 233 on the hippocampal long-term potentiation. The percentage of the baseline fEPSP slope at the last 10 min at each phase was averaged. Data are presented as mean ± SEM (n = 4). *p = 0.000 compared with the baseline phase; ^#^*p* = 0.000 compared with the LTP phase.

The hippocampal postsynaptic response was evaluated by determining the LTP magnitude, which is a popular model for examining synaptic plasticity. Following LTP induction, the perfusion of 10 µg/mL concentration of ECa 233 significantly enhanced the CA1 postsynaptic response. Compared with that by the other substances, the response further elevated significantly by ECa 233 (10 µg/mL) after being washed out (Figure 3). The initial significance was observed at 65 min after the perfusion (35 min after HFS) [273.71 ± 56.66%, F_(3,15)_ = 3.734, *p* = 0.042]. The LSD *post-hoc* test revealed that only the fEPSP level of the 10 µg/mL ECa-treated hippocampal slices robustly increased (*p* = 0.037, *p* = 0.008, *p* = 0.041) compared with the fEPSP levels of the ACSF-treated (181.26 ± 38.19%), 0.01% DMSO-treated (148.74 ± 5.40%), and 100 µg/mL ECa-treated (182.17 ± 18.61%) hippocampal slices. In addition, a significant interaction existed between the effects of phase and the experimental group on fEPSP slopes as follows: [group effect: F_(3,48)_ = 186.082, *p* = 0.000; phase effect: F_(2,48)_ = 139.463, *p* = 0.000; group x phase interaction: F_(6,48)_ = 49.672, *p* = 0.000] (Figure 4). Throughout the recording time, only the 10 µg/mL ECa-treated hippocampal slices significantly exhibited the gradual increase of the fEPSP response (*p* = 0.000). Thus, ECa 233 at 10 µg/mL concentration significantly enhanced the synaptic plasticity. Furthermore, ECa 233 acted locally and had a rapid onset once present in the area, gradually increasing the synaptic response. Although ECa 233 was already removed, its action persisted further, suggesting that once the machinery has been triggered, the effect can persist and does not require the active molecules of ECa 233 to maintain the effect.

## Discussion

In our previous *in vivo* study, ECa 233 had an inverted U-shaped response curve on hippocampal synaptic plasticity. The effective dose of ECa 233 (30 mg/kg) enhanced the expression of plasticity-related proteins, the hippocampal LTP, and also the memory retention, as observed in the behavioural test (Boondam et al. 2019). However, the effect of orally administered *C. asiatica* extract depends on many absorption-influencing factors, such as intestinal metabolism and efflux transporters (Anukunwithaya et al. 2017a).

Madecassoside, which is an active constituent of ECa 233, also acts as a substrate for P-glycoprotein and multidrug-resistant protein 2, mediating the drug efflux and reducing drug absorption, respectively (Anukunwithaya et al. 2017a). In humans, the extract is metabolized to triterpenic acid metabolites by β-glycosidases from gut microbiota, which directly hydrolyse the glycosidic bonds of plant glycosides (Rowland et al. 2018); therefore, the constituents derived from the extract in the human blood circulation were triterpenoid glycosides as the parent compounds (madecassoside and asiaticoside), and triterpenic acid metabolites (madecassic acid and asiatic acid). Following the oral administration of ECa 233 at 250 mg/kg dose for 7 days, the C_max_ ratio in plasma between madecassoside and madecassic acid was approximately 1:10, whereas that between asiaticoside and asiatic acid was approximately 1:9; thus, triterpenic acid metabolites have considerably higher plasma concentrations than the parent compounds in humans (Songvut et al. 2019).

Furthermore, the triterpenic acid metabolites have neuroprotective and anxiolytic effects on learning and memory (Nasir et al. 2011; Sirichoat et al. 2015; Chaisawang et al. 2017), suggesting that the active constituents producing the learning-and-memory effect might be triterpenic acid metabolites instead of triterpenoid glycosides, which are the main constituents of *C. asiatica* extract. Hence, we wanted to confirm the exact active constituents by demonstrating and emphasizing the potency of triterpenoid glycosides in ECa 233 (the standardized *C. asiatica* extract containing 85% of triterpenoid glycosides and <1% of triterpenic acid metabolites) on synaptic enhancement. This study investigated the hippocampal synaptic enhancement by directly perfusing ECa 233 on acute hippocampal slices. This method eliminates absorption-influencing factors and gut metabolism and also reduces the period of ECa 233 onset; thus, we can examine the direct potency of ECa 233.

Moreover, this direct perfusion method aimed to delineate that the main active compounds of ECa 233 acting on the hippocampus are triterpenoid glycosides (madecassoside and asiaticoside), not their metabolites. We selected the tested concentrations of ECa 233 from an *in vitro* study demonstrating the neuritogenic effects of ECa 233 on human neuroblastoma cells (Wanakhachornkrai et al. 2013).

The communication between neurons requires normal basal synaptic transmission, which starts when Ca^2+^ ions influx into the cells, triggering the release of neurotransmitters from the presynaptic membrane. Memory formation requires the release of glutamate, which is a major excitatory neurotransmitter in the nervous system. These glutamates bind to specific receptors such as ionotropic glutamate receptor (NMDAR and AMPAR) and metabotropic glutamate receptor (mGluR) on the postsynaptic membrane. After binding, excitatory postsynaptic potentials (EPSPs) are generated. The binding of glutamate to NMDARs also induces Ca^2+^-dependent signalling cascades, which mediate many neurotrophic protein transcriptions and synaptic plasticity (Sweatt 2010). As mentioned, the LTP model is generally used to study synaptic plasticity (Hölscher 1999), representing the mechanism of the episodic memory encoding and consolidation in the hippocampus (Clopath 2012). Therefore, LTP magnitude enhancement indicates the increased capability of hippocampal synapses to encode and consolidate information, which is important for learning and memory.

According to our study results, the basal excitatory synaptic transmission was not altered by the direct perfusion of ECa 233 on acute hippocampal slices at any concentrations, and only ECa 233 at 10 µg/mL concentration enhanced the hippocampal LTP magnitude after HFS; even after ECa 233 removal, the LTP magnitude further increased significantly. The pattern of ECa 233-induced LTP is similar to that of BDNF-induced EPSP responses (Kang et al. 1997; Bramham and Messaoudi 2005); it is also similar to the pattern of LTP after the chronic ECa 233 treatment in normal rats in our previous study (Boondam et al. 2019). Upregulation of several NMDAR subunits, PSD-95, p-CaMKII, p-PKACβ, p-CREB, BDNF, and TrkB, after the administration of *C. asiatica* extract, including its parent compounds, could promote the expression of hippocampal LTP and synaptic formation (Lin et al. 2004; Yin et al. 2015; Boondam et al. 2019). Especially, the upregulation of NMDAR subunits is necessary for LTP formation in the Schaffer collateral pathway of the hippocampus (Tsien et al. 2012). Moreover, low dosages of *C. asiatica* extract elevated the expression of AMPAR subunit, which is essential for stabilizing the synaptic strength (Binti Mohd Yusuf Yeo et al. 2018). After TrkA or TrkB receptor activation, both asiaticoside and madecassoside had more potency for the sustained activation of MEK/ERK-CREB phosphorylation and PI3K/Akt pathways than asiatic acid and madecassic acid (Nalinratana et al. 2018). Molecular activation promoted the neurite outgrowth, which is a necessary process for memory enhancement.

When the activity of asiaticoside is prolonged, the glutamate release at the presynaptic membrane might be altered, further mediating the synaptic transmission. The long-term asiaticoside treatment in mice promotes the upregulation of many hippocampal proteins, such as synapsin I-a presynaptic protein (which mediates neurotransmitter release), PSD-95, and BDNF (a neurotrophic factor regulating the expression of many synaptic proteins) (Luo et al. 2015). Conversely, asiatic acid does not alter the synaptic transmission and the hippocampal LTP (Mook-Jung et al. 1999). It also suppresses glutamate release at the presynaptic membrane by inhibiting the influx of Ca^2+^ ions (Lu et al. 2019). However, the memory-enhancing effect of asiatic acid might be associated with other mechanisms, such as the antioxidant mechanism.

In the present study, the synaptic response after ECa 233 administration still exhibited an inverted U-shaped response curve. At a high concentration level, the synaptic response decreased. This response might be related to and explained by the anxiolytic effect of ECa 233, considering that *C. asiatica* extract and ECa 233 have demonstrated the anxiolytic effect dose-dependently (Wijeweera et al. 2006; Awad et al. 2007; Wanasuntronwong et al. 2012). Thus, the higher doses will express more anxiolytic effect. Additionally, *C. asiatica* extract can act as a GABA agonist and promote the activity of glutamic acid decarboxylase, an enzyme associated with GABA synthesis (Awad et al. 2007). The increased GABA activity by anxiolytic drugs, such as benzodiazepines, could inhibit hippocampal LTP (del Cerro et al. 1992).

Moreover, the oral administration of *C. asiatica* extract depends on many absorption-influencing factors. The effects of ECa 233 could possibly be related to some absorption-influencing factors, such as intestinal metabolism and efflux transporters (Anukunwithaya et al. 2017a). As observed, the oral administration of ECa 233 demonstrated a slow onset of action, possibly because of its poor oral bioavailability and the large molecular weight of its parent compounds (Anukunwithaya et al. 2017b; Khemawoot et al. 2018); hence, the distribution of the compounds to various organs might be time dependent. According to a pharmacokinetic study, 4 h after ECa 233 administration, the distribution of the parent compounds in the brain tissues by the oral route was slightly lower than that by the IV route (Anukunwithaya et al. 2017b). Moreover, the elimination of half-life of the parent compounds after oral administration was shorter than that after IV administration (Khemawoot et al. 2018). The direct perfusion of ECa 233 on acute hippocampal slices would demonstrate the exact potency of ECa 233 on the brain tissues.

**Figure 5.**
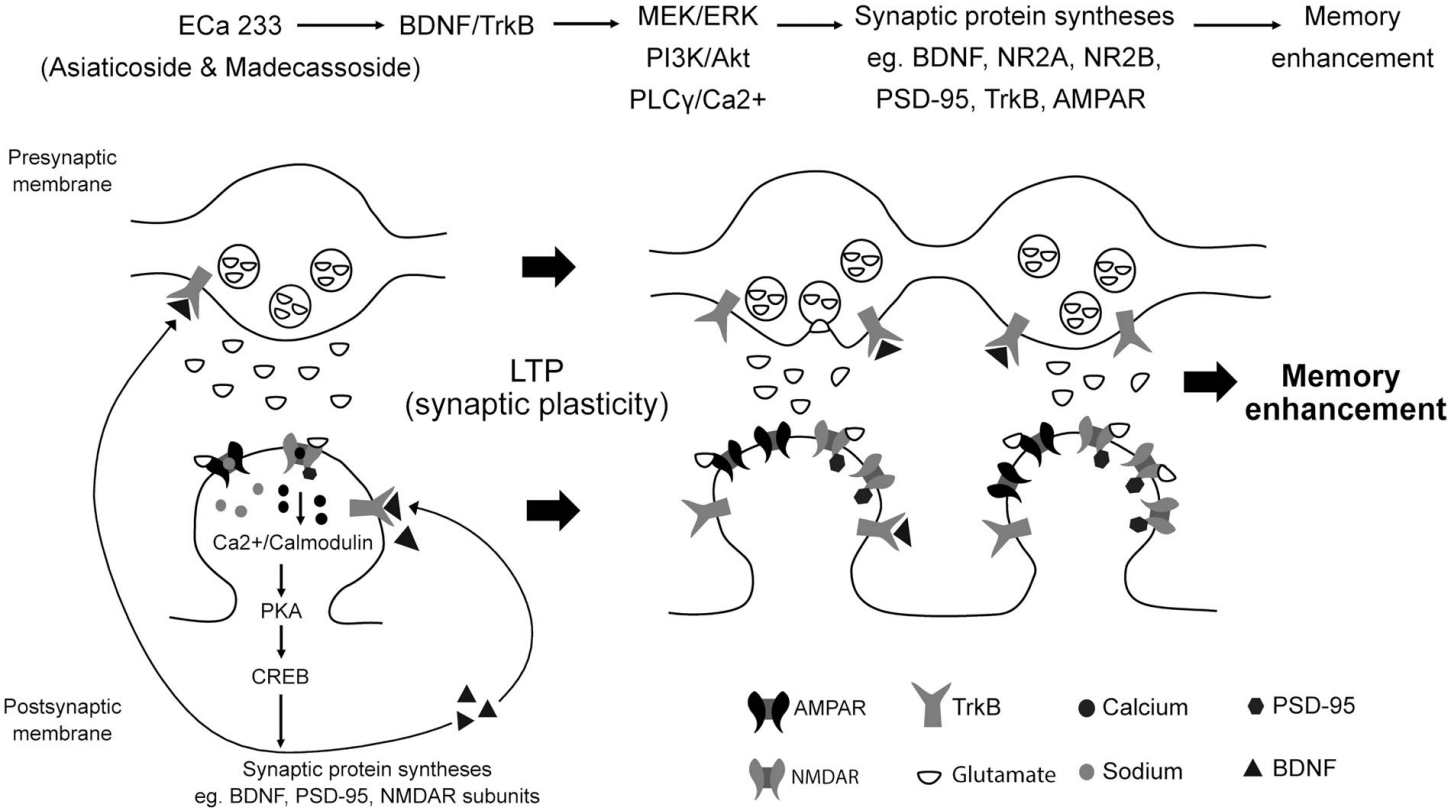
Schematic representation of the possible mechanisms of ECa 233 on synaptic plasticity. Low doses of ECa 233 via the main effects of asiaticoside and madecassoside might activate the MAPK/ERK, PI3K/AKT, and PLCγ pathways, leading to many protein syntheses, including BDNF, NMDAR subunits (NR2A and NR2B), AMPAR, PSD-95, TrkA, TrkB, and synapsin I. Moreover, ECa 233 might promote the phosphorylation activity of p-CaMKII, p-PKACβ, and p-CREB. Then, BDNF binds to TrkB at both pre- and postsynaptic membranes. These bindings trigger three signalling cascades (PLCγ/Ca^2+^, PI3K/Akt, and MEK/ERK) mediating synaptic transmission (glutamate release elevation), LTP, and neurite outgrowth. The enhanced expression of plasticity-related proteins mediates the enhancement of synaptic plasticity, which is exhibited as the hippocampal LTP. Finally, all cellular changes that are part of the basic mechanism of learning and memory might be expressed as memory enhancement.

Our study could confirm and emphasize that ECa 233 has a strong synaptic plasticity-enhancing effect. The potency of the ECa 233 effect most likely depends on the action of parent compounds (triterpenoid glycosides). Both of the triterpenoid glycosides, namely, asiaticoside and madecassoside, could play a role in a nootropic activity because both constituents have not been metabolized to derivative forms. Both of them enhance the hippocampal LTP, which is the basic characteristic of memory formation. However, the BDNF-TrkB downstream signalling pathways still need to be confirmed as an underlying mechanism for synaptic enhancement (Figure 5).

## Conclusions

The parent compounds of ECa 233, namely, asiaticoside and madecassoside, play an important role in synaptic plasticity, which is the cellular mechanism of learning-and-memory formation. However, further investigation is needed to elucidate the possible mechanisms for synaptic enhancement associated with the action of BDNF/TrkB signalling pathway. Hence, this study provides a new information essential for the development of a new neurodegenerative drug using ECa 233 for the treatment of memory impairment.
